# Genome editing in mouse spermatogonial stem cell lines targeting the Tex15 gene using CRISPR/Cas9

**DOI:** 10.3389/fvets.2025.1599598

**Published:** 2025-05-14

**Authors:** Suheyla Yeşilbostan, Mustafa Yenal Akkurt, Sinan Özkavukçu, Oǧuz Kul, Bengi Çınar

**Affiliations:** ^1^Independent Researcher, Frankfurt, Germany; ^2^Department of Genetics, Faculty of Veterinary Medicine, Ankara University, Ankara, Türkiye; ^3^Department of Histology and Embryology, School of Medicine, Ankara Medipol University, Ankara, Türkiye; ^4^Bahçeci Health Group, Ankara, Türkiye; ^5^Department of Pathology, Faculty of Veterinary Medicine, Kırıkkale University, Kırıkkale, Türkiye

**Keywords:** CRISPR/Cas9, genome editing, spermatogonial stem cells, Tex15 gene, transfection optimization, animal reproductive biology

## Abstract

CRISPR/Cas9-mediated DNA endonuclease technology has been extensively utilized to introduce targeted genomic mutations for investigating biological processes across various cell types and organisms. In spermatogonial stem cells (SSCs), CRISPR/Cas9 has proven to be an effective tool for elucidating the genetic mechanisms underlying spermatogenesis and infertility. Additionally, it holds potential applications in disease prevention, transgenic animal production, and genetic improvement of livestock. This study aimed to optimize the lipid-based transfection of a lentiviral plasmid vector into SSCs by targeting the Tex15 gene, which is associated with infertility in humans, using CRISPR/Cas9. The efficiency of genome editing was assessed by detecting frameshift indel mutations starting from c.959C in exon 1 of the Tex15 gene using mutation site enzyme cut analysis, sanger sequencing, and *in silico* analyses. The highest transfection efficiency was achieved with a 1:3.5 DNA:DNAfectin ratio, which was identified as the optimal condition for SSC transfection. CRISPR-Cas9 editing in a monoclonal cell line derived from a single cell yielded high efficiency (model fit *R* = 0.97). Sequence analysis revealed two possible indel variants, indicating possible heterozygous biallelic editing within the same genome. Our findings demonstrate the potential of SSC-mediated genome editing for generating transgenic animals, enhancing productivity in livestock, and advancing novel therapeutic strategies for genetic disorders in animals and human male infertility.

## 1 Introduction

Spermatogonial stem cells (SSCs) constitute the adult stem cell population within the testis, possessing the ability for self-renewal, and differentiation into precursor cells of spermatozoa, thereby forming the basis of spermatogenesis (1, 2). The regulation of SSC renewal and differentiation is regulated intrinsically by gene expression and extrinsically by signals from the surrounding niche (3, 4).

In rodents, SSCs reside along the basal membrane of seminiferous tubules as isolated A single (As) spermatogonia, which undergo mitotic divisions to either self-renew and maintain the stem cell pool or differentiate into A paired (Apr) spermatogonia. Apr spermatogonia further divide and clonally expand into aligned (Aal) spermatogonia through cytoplasmic bridges. These Aal cells eventually differentiate into A1 spermatogonia, followed by sequential divisions into A2, A3, A4, Intermediate, and B spermatogonia, the latter giving rise to primary spermatocytes through a final mitotic division (5–7).

Manipulation of SSCs offers promising opportunities for understanding germline regulatory mechanisms, developing germline modifications, and advancing new therapeutic techniques. However, due to the low abundance and challenges in precise characterization of SSCs, research on their biology and role in male germline regulation has been a long-standing challenge (8). One of the potential clinical applications of SSCs lies in germline gene therapy, which holds promise for correcting spermatogenic failure and preventing congenital genetic disorders that result in life-threatening conditions. To develop these therapeutic approaches, establishing *in vitro* SSC culture systems is a prerequisite (4, 7, 9).

Murine SSC cultures serve as a model system for optimizing conditions applicable to human and other mammalian SSCs (10, 11). Spermatogenesis is regulated by ~1,500–2,000 genes (12), and mutations in these genes are a major cause of male reproductive disorders (13). Razavi et al. (12) analyzed gene expression variations in human and murine infertility cases, identifying multiple genes associated with male infertility. Among these, Tex15 mutations have been reported to cause testicular atrophy and meiotic arrest at the early spermatogenic stage in mice (14). Tex15 is located on mouse chromosome 8, spans 15 kb, and consists of four exons encoding a 2,785-amino acid protein expressed exclusively in the testis and ovary (15). Tex15 expression is dynamic throughout spermatogenesis, with abundant transcripts in spermatogonia, early spermatocytes, and post-meiotic germ cells (16). In Tex15 knockout mice, early meiotic arrest and loss of post-meiotic germ cells have been observed, with no effect on female fertility (15, 17, 18). Additionally, a mutation in exon 1 of the Tex15 gene has been associated with infertility in a Turkish family, leading to progressive sperm count reduction over time (19).

CRISPR/Cas9 technology has emerged as an efficient and widely used tool for targeted genetic modifications (20), surpassing earlier gene-editing strategies such as ZFN and TALEN (21–23). Unlike these protein-based approaches, CRISPR/Cas9 enables flexible targeting by simply altering the guide RNA sequence, allowing precise genetic modifications (24). The delivery systems for CRISPR components, particularly the Cas9 protein and guide RNA, are critical in determining the efficiency and specificity of gene editing. Various delivery methods have been explored based on the target cell type—each exhibiting its own advantages and limitations (25, 26). In the context of spermatogonial stem cells, specific studies highlighted the efficiency of CRISPR/Cas9-mediated genome editing using this precise delivery system. For instance, Chapman et al. (27) reported successful targeted gene modifications in rat germline stem cells, involving HDR (homology-directed repair) techniques while another notable delivery method is the use of plasmid DNA (28, 29).

In this study, we applied CRISPR/Cas9-mediated gene editing in a type B SSC line to determine whether the Tex15 gene could be effectively targeted. Following CRISPR-Cas9 application, we assessed cell viability and editing efficiency using mutation site enzyme digestion, DNA sequencing, and *in-silico* analyses to confirm the modifications leading to knockout mutations.

This study aims to optimize the lipid-based transfection of a lentiviral plasmid vector for SSCs and evaluate CRISPR/Cas9-mediated Tex15 gene silencing. We hypothesize that an optimized transfection protocol will enhance gene-editing efficiency, providing a robust platform for SSC-mediated genome modification, transgenic animal production, and reproductive biotechnology advancements.

## 2 Materials and methods

### 2.1 Spermatogonial stem cell culturing

A commercial mouse spermatogonial stem cell (SSC) line (ATCC CRL: 2053) was used in this study. The cells were stored at −80°C until use and maintained in fresh culture passages in liquid nitrogen. All cell culture experiments were conducted in the Genetics Department's Cell Culture Laboratory. Dulbecco's Modified Eagle Medium (DMEM) was used as the basal medium, supplemented with 10% fetal bovine serum (FBS) and 1% penicillin-streptomycin. The vial containing SSCs was thawed at 37°C in a water bath and centrifuged at 125 × g for 5 min. The supernatant was discarded, and the cell pellet was resuspended in 3 mL of fresh growth medium and transferred into a 25 cm^2^ culture flask. Cells were incubated at 37°C in a 5% CO_2_ environment and subcultured upon reaching 90% confluence using trypsinization. The morphology and proliferation of SSCs were monitored using an inverted phase-contrast microscope (Leica DM IRB Inverted Microscope, Japan).

### 2.2 Subculture and cryopreservation

Cells were washed with phosphate-buffered saline (PBS) (Ambresco, Cat. No: E404) before adding 3.0 mL of 0.25% trypsin-0.53 mM EDTA solution. The culture was incubated at 37°C for 5–15 min until cells detached, followed by centrifugation at 2,500 rpm for 3 min. The resulting cell pellet was resuspended in 1 mL of growth medium. Ten microliter cell suspension was counted using a Makler chamber and seeded into 25 cm^2^ and 75 cm^2^ flasks based on their density. Data from a single 25 cm^2^ culture flask were used to calculate the doubling time of cultured spermatogonial stem cells according to Equation 1, where DT represents the doubling time and CTS refers to the cell culture duration.

Doubling time calculation:


(1) $$\begin{array}{l} DT=CTS\times \frac{\log\left(2\right)}{\log\left(final\;cell\;count\right)-\log\left(starting\;cell\;count\right)} \end{array}$$

To prevent differentiation due to repeated passages, SSC cultures were frozen in liquid nitrogen after each passage. The initial SSCs were expanded for the first five passages, and only these early-passage cells were used in genetic experiments. Cells were also cryopreserved for future studies by freezing them in liquid nitrogen. In order to confirm that no morphological differentiation occurred in passaged cells, they were attached to slides via cytocentrifugation, fixed with methanol, and stained with toluidine blue for 30 min.

Following cell counting, cells were supplemented with dimethyl sulfoxide (DMSO) at a final concentration of 10% and initially stored at −80°C for 24 h. Finally, the cells were transferred to liquid nitrogen (−196°C) for long-term storage.

### 2.3 Antibiotic dose optimization

To determine the optimal concentration of puromycin for selecting successfully transfected cells, a cytotoxicity assay was performed. SSCs were plated in a 96-well plate at a density of 8 × 10^5^ cells/10 mL per well and treated with different puromycin concentrations (ranging from 5.0 μg/mL to 0.01 μg/mL) over 48 h. The selection of puromycin concentration was based on the minimum dose required to eliminate 95% of non-transfected cells. A control group without puromycin was included.

Cell viability was assessed using an MTT assay, where 10 μL of MTT reagent (5 mg/mL) was added to each well, followed by incubation at 37°C for 4 h to allow for the formation of formazan crystals. The reaction was terminated by adding 100 μL of DMSO, and absorbance was measured at 570 nm using a Spectra Max i3 spectrophotometer. Each concentration was tested in triplicate and applied on two separate days following the same procedure.

Additionally, morphological changes in the cells were examined under an inverted phase-contrast microscope (Leica DM IRB, Japan). The LC50 value (the lethal concentration required to kill 50% of the cells) was determined based on a dose-response curve.

### 2.4 Plasmid transformation and purification

In this study, a non-viral plasmid-mediated liposome transformation method was applied.

The sgRNA target sequences were pre-cloned into the pLenti-U6-sgRNA-SFFV-Cas9-2A-Puro vector, which was obtained as part of the Tex15 sgRNA CRISPR-Cas9 All-in-One vector set and used in its supercoiled circular form for transfection (ABM, Cat. No: K3130705). A sham control group was designed using scrambled lentivector plasmids (ABM, Cat. No: K010), which did not contain a specific target region.

The Tex15 specific sgRNA sequences, PAM sequences and their nucleotide positions are provided in Table 1. The positions of the target sites within exon 1 of the gene were verified using the Ensembl genome database (GRCm38.p6 version, Transcript ID: ENSMUST00000009772.8) (30).

**Table 1:** The positions of the target sites within exon 1 of the Tex15 gene.

**sgRNA ID**	**Strand**	**Genpmic start position**	**sgRNA sequence (5^′^-3^′^)**	**PAM**	**PAM start position**
Target 1	Sense	c.939	ACTTGTAGCAACGACTCTCA	GGG	c.959
Target 2	Sense	c.1,328	TGAACTATATCGTGCAGCAT	TGG	c.1348
Target 3	Sense	c.1,700	TCGTGTTAAAGATGGTGTGC	AGG	c.1720

Strand, indicates the orientation of the sgRNA with respect to the coding (sense) strand of the Tex15 gene; Genomic Start Position, the position of the first nucleotide of the sgRNA target site within the exon 1 sequence. PAM, protospacer Adjacent Motif sequence required for SpCas9 recognition; PAM Start Position, the genomic position of the first nucleotide of the PAM sequence immediately downstream of the sgRNA site.

Competent E. coli DH5α cells were used for transformation. The bacterial suspension was thawed on ice for 15 min, and 1 μL of plasmid DNA (10 ng/μL) was added and gently mixed. The mixture was incubated on ice for 30 min, followed by heat shock at 42°C for 2 min, then immediately transferred back to ice for 2 min. After adding 150 μL of LB broth, the culture was incubated at 37°C with shaking at 220 rpm for 1 h. The transformed bacteria were then spread onto ampicillin-containing LB agar plates and incubated overnight at 37°C.

### 2.5 Plasmid purification

Colonies selected from transformation plates were cultured overnight in 5 mL LB medium containing ampicillin (100 μg/mL) at 37°C, 220 rpm. Plasmids were purified using the GeneJet Plasmid Miniprep Kit (Thermo Scientific, Cat. No: K0502). The purification steps involved alkaline lysis, neutralization, and ethanol precipitation, followed by elution in 50 μL of pre-warmed elution buffer (70°C). DNA concentrations and purity ratios (260/280 and 260/230) were measured using a Thermo Fisher ND-2,000 NanoDrop spectrophotometer.

To confirm successful plasmid transformation, the isolated plasmids were digested using the KpnI restriction enzyme (ABM, Cat. No: E054), which recognizes the sequence GGTAC∧C. Each reaction contained 5 μL of DNA (300 ng), 2 μL of 10 × enzyme buffer, 0.25 μL of KpnI enzyme, and 17.75 μL of ddH_2_O, making a total reaction volume of 25 μL. Samples were incubated at 37°C for 30 min and analyzed using 1% agarose gel electrophoresis at 100 V for 20 min. The expected band sizes for pLenti-U6-sgRNA-SFFV-Cas9-2A-Puro were 5.5 kb and 6.3 kb, confirming correct plasmid digestion.

### 2.6 Transfection optimization

To achieve maximum transfection efficiency with minimal cytotoxicity, transfection conditions were optimized by varying the DNA and DNAfectinTM2100 (ABM, Cat. No: G2100) concentrations along with cell density (Table 2). Cells were seeded at a density of 1–2.5 × 10^5^ cells per well in a 6-well plate with 2.0 mL of appropriate growth medium. Cells were incubated at 37°C in 5% CO_2_ until they reached 70%−90% confluency.

**Table 2:** Optimization of DNA concentration and DNAfectin amounts for transfection along with cell density.

**Transfection conditions**	**Ratios**					
DNA (μg): DNAfectin (μL)	1:1	1:2.5	1:3.5	1:5	1:7.5	1:10
Cell : DNA-DNAfectin mix	2:2	2:5	2:7	2:10	2:15	2:20

DNA-DNAfectin complexes were incubated at room temperature for 20 min. The transfection complex was then added to 800 μL of serum- and antibiotic-free growth medium. The cells were washed with PBS, and the medium was replaced with 1 mL of transfection mixture per well. Cells were incubated at 37°C in 5% CO_2_ for 6–8 h, after which the transfection medium was replaced with 2 mL of complete growth medium, and the cells were incubated overnight. After 24 h, cells were trypsinized and counted using a hemocytometer. A 1:10 dilution was prepared, and cells were monitored for 1–2 days. To select successfully transfected cells, puromycin (2 μg/mL) was added 48 h post-transfection. Non-transfected cells, which were sensitive to puromycin, were eliminated. Only puromycin-resistant cells carrying the plasmid were expected to survive, confirming successful transfection.

Transfection experiments were performed using three target sgRNA sequences for Tex15 gene silencing, along with control groups:

1. CRISPR-Cas9 groups: three groups transfected with sgRNAs targeting different sites on Tex15 (Target 1, Target 2, Target 3).
2. Sham (Scrambled) group: cells transfected with a scrambled sgRNA vector that does not target a specific gene, used as a negative control.
3. Control group: cells cultured without any transfection.
4. DNAfectin-only group: cells treated with DNAfectin alone to observe any potential cytotoxic effects.

### 2.7 Validation of CRISPR-mediated transfection experiments

#### 2.7.1 DNA isolation from cells

Genomic DNA was isolated using the Genomic Cleavage Detection Kit (ABM, Cat. No: G932) following the manufacturer's instructions. Cells were detached using trypsin and counted, ensuring a final pellet of 5 × 10^4^ cells was prepared. The required volume of DNA was extracted for PCR amplification, while the remaining DNA was stored at −20°C. The concentration and purity of isolated DNA were assessed using a Thermo Fisher ND-2,000 NanoDrop spectrophotometer.

#### 2.7.2 PCR amplification and mutation enzyme digestion analysis

The oligonucleotide sequences used for PCR amplification of the Tex15 target regions are presented in Table 3. The PCR cycling began with an initial denaturation phase at 95°C for 10 min, followed by 40 cycles consisting of denaturation at 95°C for 30 s, annealing at a gradient temperature range between 54°C and 66°C for 30 s, and extension at 72°C for 1 min. A final extension step at 72°C for 5 min ensured the completion of all amplified DNA fragments. PCR amplification products were analyzed by loading 5 μL of PCR product onto a 1% agarose gel. Electrophoresis was performed at 120 V for 30 min, and bands were visualized using a UV transilluminator. To confirm gene editing, mutation enzyme digestion was performed according to the Genomic Cleavage Detection Kit's (ABM, Cat. No: G932) protocol. Electrophoresis was performed at 110 V for 30 min, and DNA bands were visualized under UV light. The presence of cleaved bands indicated successful CRISPR editing.

**Table 3:** Target-specific primer sequences.

**Target region**	**Primer direction**	**Primer sequence (5'−3')**
Target 1	Forward	GAGATGGGTCCTTTCAGCTC
	Reverse	GGCTCTCATCATTCCCGTAT
Target 2	Forward	AGTGATGTTTTGCCATTGGA
	Reverse	CTGGAAGGCATCAGACAAAA
Target 3	Forward	CAGCTGCCATTGACATCTCT
	Reverse	CCCAATCAATCCGAGACTTT

#### 2.7.3 Sanger sequencing

To verify the intended genome editing via Sanger sequencing, internal primers were designed using Primer Web Version 4.1.0. Target 1 and 2 shared the same forward primer SeqF: 5′-CCATCAGCACAGAAGACAGC-3′ and SeqR for Target 3: 5′-TGGTGCATGCCTTTGTTCTA-3′. Before loading onto the ABI sequencing system, PCR products were purified using the ZYMO DNA Sequencing Clean-Up Kit (Cat. No: D4050) according to the manufacturer's recommendations. Sanger sequencing was performed using the Big Dye Terminator v3.1 Cycle Sequencing Kit (Thermo Fisher), and sequencing was carried out using an ABI Prism 310 Genetic Analyzer. Prior to sequencing, PCR products were purified using the QIAquick Purification Kit (Qiagen, Cat. No: 28106) according to the manufacturer's recommendations to remove contaminants.

#### 2.7.4 Establishment of monoclonal cell lines

In cases of heterogeneous cell populations following transfection, single cells were isolated using limiting dilution cloning to establish monoclonal cell lines. For the isolation of monoclonal cell lines, 4,000 cells were seeded in the A1 well of a 96-well plate. The cell count was determined using a Beckman Coulter CytoFlex flow cytometry system, and appropriate dilutions were made to distribute single cells across wells. Serial 1:2 dilutions were performed until a single cell was isolated in the H12 well. The cells were cultured for 15 days, monitored for proliferation, and expanded colonies were subjected to PCR and sequencing analysis to confirm successful editing.

#### 2.7.5 Determination of knockout efficiency

The proportion of knockout (KO) alleles in the cell population was determined using the ICE (Inference of CRISPR Edits) web-based analysis tool (31). The ICE algorithm functions by uploading a control ab1 file, an experimental ab1 file, and the guide RNA sequence. The algorithm evaluates data quality and identifies potential editing events. It then applies a regression fitting model to determine the presence of edited sequences and calculates the editing efficiency (32). The ICE algorithm compared the sequence reads from the transfected population to the wild-type control and estimated the indel formation rate. Sequencing results of Target 1 were analyzed to determine the percentage of knockout alleles in the cell population.

## 3 Results

### 3.1 Spermatogonial stem cell culture

**Figure 1:**
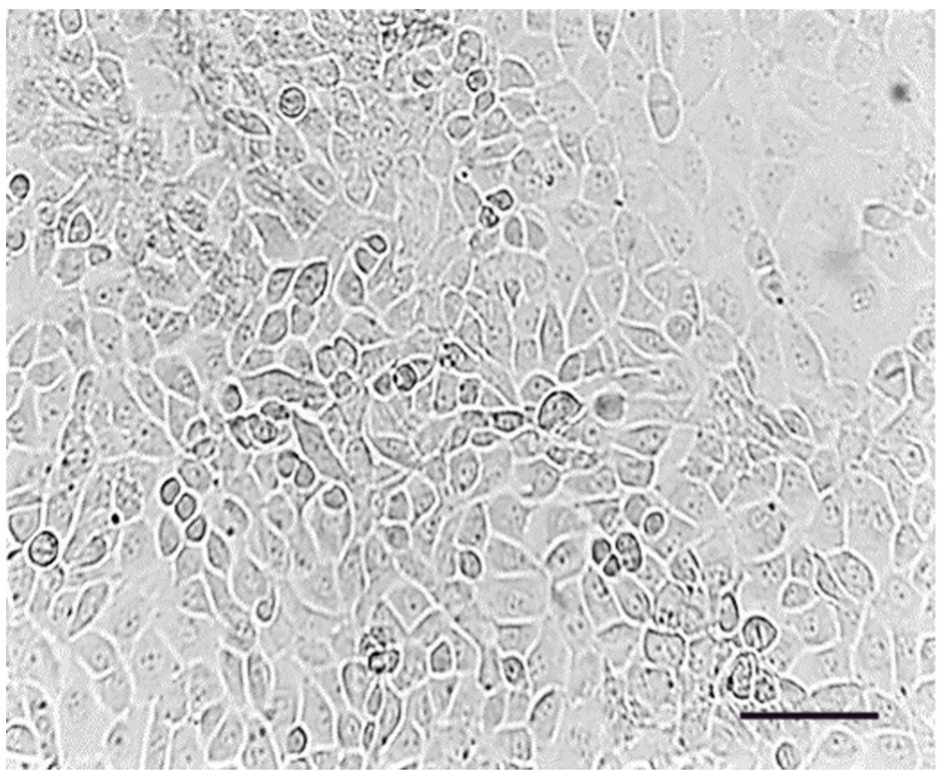
Passage (0), spermatogonial stem cells adhered to the bottom of the flask. Cell line on day 7; Scale bar: 200 μm.

**Figure 2:**
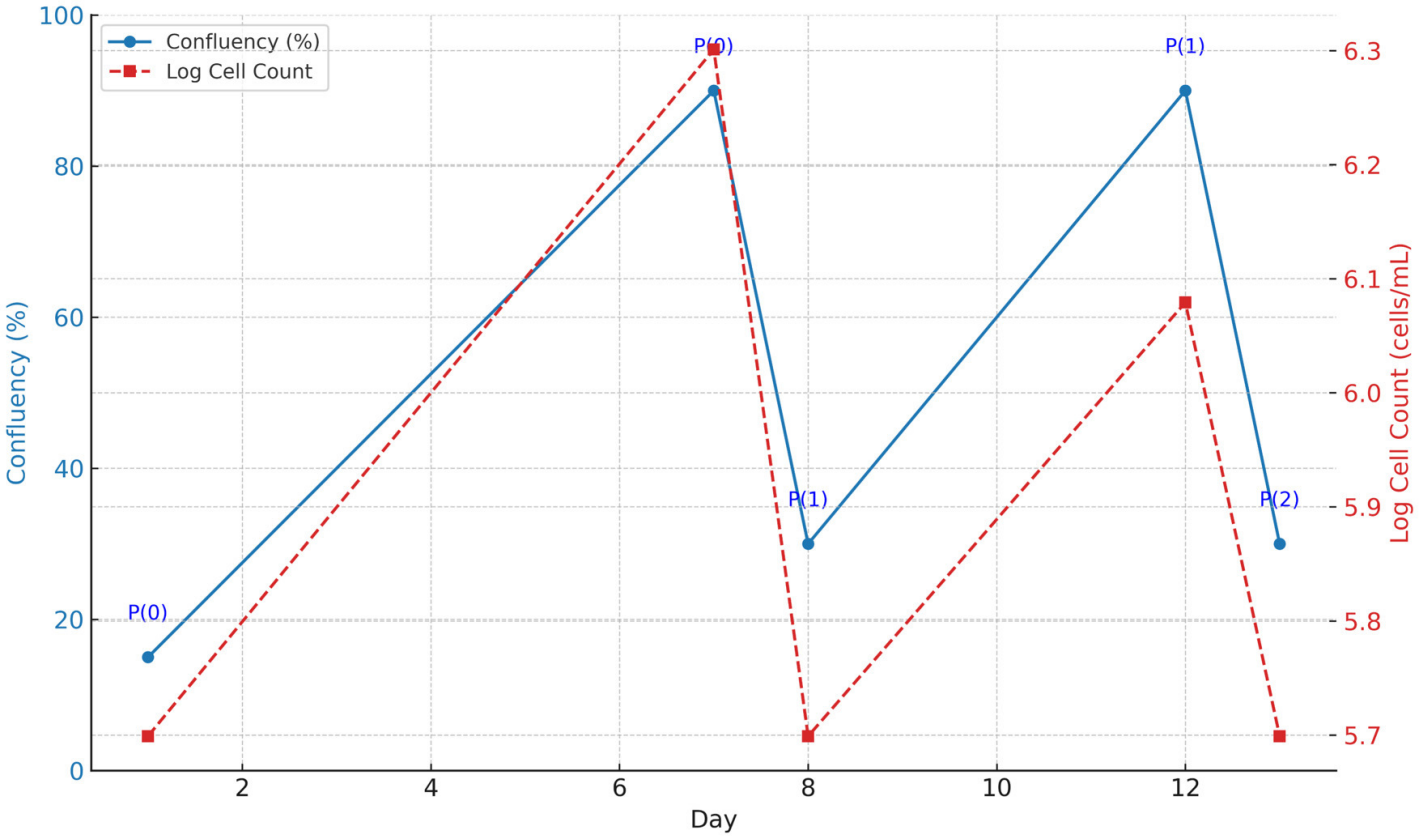
Cell growth progression from Passage 0 (P0) to Passage 2 (P2). The blue solid line indicates the confluency (%) of the cells over time, displayed on the left y-axis. The red dashed line represents the logarithmic cell count (cells/mL), displayed on the right y-axis. Passage points (P0, P1, P2) are annotated along the confluency curve. The graph illustrates consistent cell proliferation after each passage.

The initial passage (P0) of Type B spermatogonial stem cells was monitored at days 1 and 7, revealing an increase in oval and fusiform-shaped cells. Cytological assessment using toluidine blue staining confirmed the presence of undifferentiated spermatogonial cells, with no fibroblast differentiation observed. The doubling time of the spermatogonial stem cell population was calculated to be 84 h based on Equation 1. The morphology and growth of cells over time were visualized using phase-contrast microscopy (Figure 1). The Figure 2 illustrates progression of cell growth and confluency at different passages.

The MTT cytotoxicity assay was performed to determine the optimal concentration of puromycin for selection. A range of concentrations (1 μg/mL–5 μg/mL) was tested over 24 and 48 h. Cell viability significantly decreased at 2 μg/mL, making it the optimal dose for selective pressure against non-transfected cells. The cytotoxicity data indicated that cell survival dropped below 10% at 3 μg/mL, while at 1.5 μg/mL–2 μg/mL, ~50% of the cells remained viable.

### 3.2 Transformation and plasmid purification

**Figure 3:**
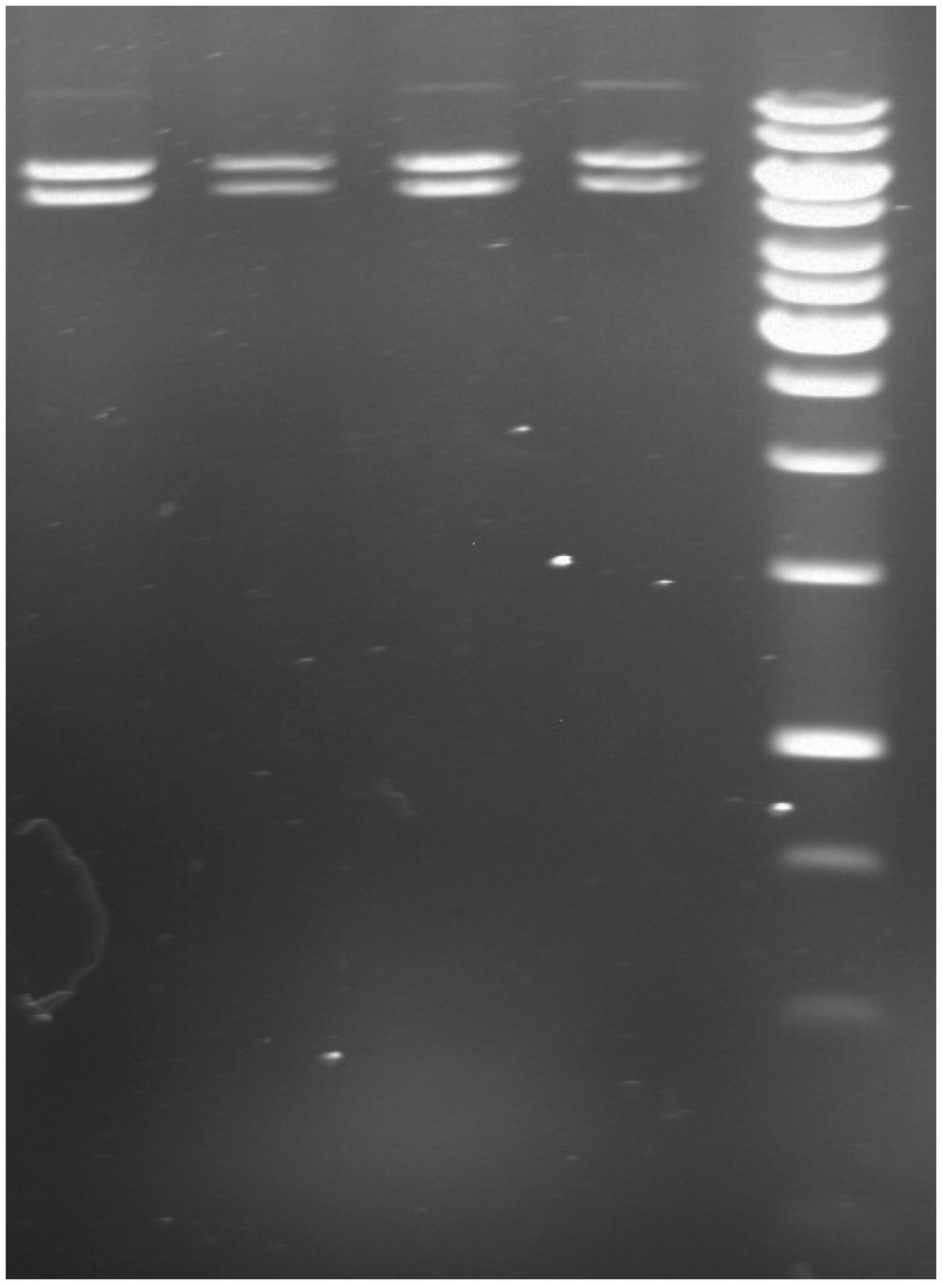
Genomic cleavage analysis following 2 min heat shock transformation. Lane 1: target 1 digestion; Lane 2: target 2 digestion; Lane 3: target 3 digestion; Lane 4: scrambled; Lane 5: 1 kb DNA ladder (Thermo Scientific, GeneRuler 1 kb DNA ladder, Cat. No: SM0311). The presence of expected fragment sizes (5.5 and 6.3 Kb) confirms the successful transformation and vector integrity.

Bacterial transformation was carried out using heat-shock protocols to introduce the pLenti-U6-sgRNA-SFFV-Cas9-2A-Puro vector into DH5α competent cells. The most efficient transformation was achieved with a 2 min heat shock, as confirmed by ampicillin-resistant colony growth. To confirm the presence of the pLenti-U6-sgRNA-SFFV-Cas9-2A-Puro vector (11.8 Kb), KpnI restriction enzyme digestion was performed on the plasmid DNA. The digestion results are illustrated in Figure 3, where the banding pattern (5.5 and 6.3 Kb) confirms the expected fragment sizes post-restriction digestion.

### 3.3 Puromycin selection

**Figure 4:**
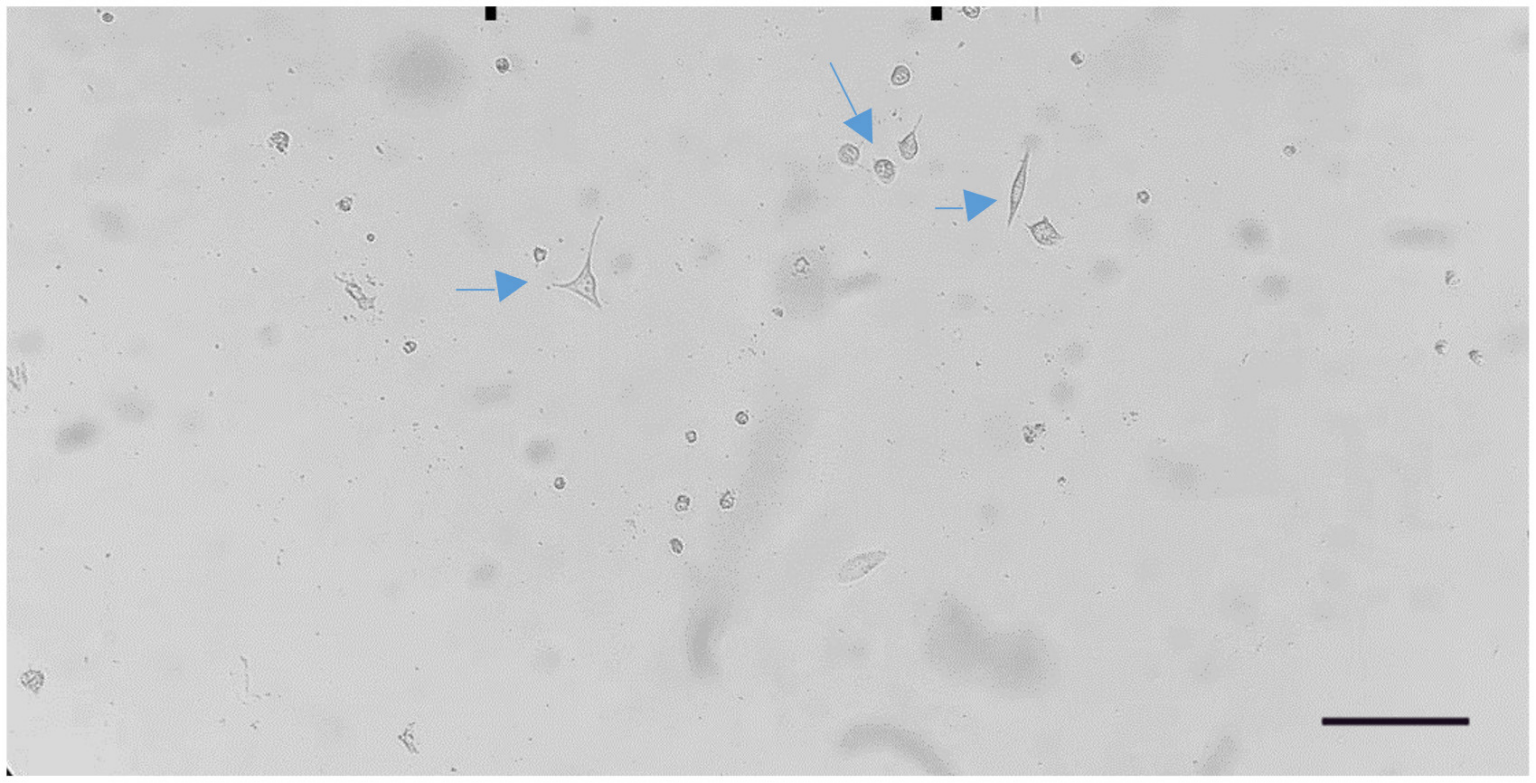
Proliferation of puromycin-resistant cells observed on day 12 after 1:3.5 DNAfectin transfection and 2 μg/mL puromycin treatment. Scale bar: 200 μm.

Cells transfected with 1:3.5 DNAfectin were treated with 2 μg/mL puromycin for 48 h, leading to the selection of puromycin-resistant colonies. These cells continued to proliferate over time, and by day 12, significant colony expansion was observed, indicating stable transfection and survival under puromycin selection (Figure 4). The increasing number of puromycin-resistant cells suggests that the applied selection pressure was effective in eliminating non-transfected cells while allowing successfully transfected clones to expand.

### 3.4 Transfection and knockout efficiency

#### 3.4.1 Genomic cleavage assay

Following transfection, DNA samples were subjected to PCR amplification and genomic cleavage analysis. The expected cleavage patterns were either faint or absent in all transfection groups, except for the positive control provided by the manufacturer. Due to the unreliable detection of transfection success using the genomic cleavage assay, all DNA samples from each group were further analyzed through DNA sequencing to confirm the presence of mutations.

#### 3.4.2 Sanger sequencing

Transfection experiments were conducted using DNAfectin at ratios ranging from 1:1 to 1:10. Successful transfection was observed only at DNAfectin ratios of 1:3.5 and 1:5 DNAfectin ratios for Target 1, as confirmed by DNA sequencing analysis, indicating a heterogeneous transfection profile. Due to this outcome, monoclonal cell line experiments were performed only on Target 1 groups.

#### 3.4.3 Monoclonal cell line establishment and knockout efficiency

**Figure 5:**
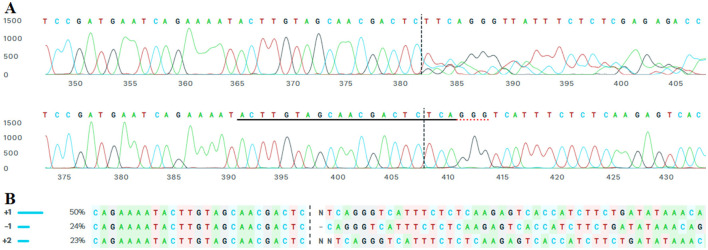
ICE-based evaluation of Target-1 region on Tex15 gene, exon 1 in the monoclonal cell line transfected with a 1:3.5 DNA: DNAfectin. **(A)** The lower trace represents the wild-type sequence **(A)**, while the upper trace shows the edited monoclonal line. The sgRNA target site is underlined; red underlines indicate the PAM sequence in the control sample, and vertical dashed lines mark the predicted Cas9 cleavage site. **(B)** Potential indels from Sanger sequencing results of the monoclonal lines.

Monoclonal cell lines were generated from successfully transfected cells through limiting dilution. After puromycin selection, single-cell isolation was performed using serial dilution in 96-well plates, ensuring that each well contained a single viable cell. These monoclonal cultures were maintained for 15 days, during which cell proliferation and stability were monitored. The monoclonal cell lines were subjected to PCR amplification and sanger sequencing, confirming the presence of the intended genetic modifications. Following to sanger sequencing, further analysis using the ICE algorithm (Synthego) provided quantitative data on the possible mutations in monoclonal population (Figure 5) and confirmed heterozygosity, suggesting a biallelic edit. CRISPR-Cas9 genome editing was performed in a monoclonal cell line derived from a single cell. The sgRNA sequence ACTTGTAGCAACGACTCTCA targeted the locus with high specificity, as indicated by a model fit value of *R* = 0.97. Indel analysis revealed a dominant sequence variant contributing 50% of the editing signal, followed by a secondary variant at 20%, and several minor variants each contributing <20%. Given the monoclonal origin from a single cell, these results represent biallelic editing events within the same genome. The presence of two major sequence variants suggests that each allele underwent a distinct indel formation, reflecting heterozygous biallelic editing. The minor variants are likely sequencing or repair-associated byproducts with limited biological relevance in this monoclonal context.

## 4 Discussion

Genome editing tools are utilized to modify specific genes, characterize gene functions, perform gene therapy, correct genetic mutations, investigate disease-specific mechanisms, and create transgenic animal models. CRISPR-Cas technology has revolutionized genome engineering by enabling scientists to edit virtually any DNA sequence in living cells and model organisms. However, the efficiency of CRISPR varies significantly across different loci and cell types.

Stem cell models are particularly powerful in establishing the mechanistic connection between genotype and phenotype. One of the most notable CRISPR applications in stem cell research was the correction of CFTR mutations in cultured intestinal stem cells from cystic fibrosis patients (33). In contrast, genetic manipulation of spermatogonial stem cells (SSCs) remains limited due to the low efficiency and complexity of existing editing techniques (34).

SSC manipulation and subsequent transplantation offer a unique opportunity to introduce desired genetic modifications. SSCs can self-renew and generate numerous spermatozoa capable of transmitting edited genes to the next generation, making them a promising candidate for producing genetically modified animals and treating heritable diseases (9). However, compared to embryonic stem cells (ESCs), SSCs exhibit slower cell cycles, lower transfection efficiency, and more challenging clonal expansion (35). These difficulties have been linked to SSC-specific genome protection mechanisms (36).

Although studies reporting CRISPR-Cas9 gene editing in SSCs and mutant rat production are limited (27, 34), they demonstrate feasibility. SSCs are typically isolated via primary culture from donor testes before transplantation into recipient mice (37). Testes contain a heterogeneous cell population that secretes various growth factors essential for spermatogenesis, including Sertoli cells, Leydig cells, and other somatic testicular cells. For SSC line establishment, these somatic cells must be removed. SSCs are rare, comprising approximately one in 3,000 cells in adult mouse testes (38), and under *in vitro* conditions, their numbers often decline while Sertoli-like and fibroblast-like cells proliferate (39).

To replicate the seminiferous tubule microenvironment for *in vitro* spermatogenesis, a mimetic environment is necessary, as meiosis and spermiogenesis naturally occur in the blood-testis barrier. Due to the complexity, cost, and time required for primary SSC culture and isolation, we utilized the commercially available ATCC GC-1 spg (CRL-2053) cell line (40) which is commonly used to investigate early spermatogenesis (41–43). The use of the commercially available GC-1 spg cell line, while advantageous for experimental reproducibility and ease of transfection, poses inherent limitations when modeling undifferentiated primary spermatogonial stem cells (SSCs). GC-1 spg cells are immortalized type B spermatogonia that have undergone SV40 large T antigen-induced transformation, and as such, may not faithfully recapitulate the gene expression profile, epigenetic status, or stemness characteristics of primary SSCs isolated from testicular tissue (40, 44). Furthermore, GC-1 cells lack the functional capacity for full spermatogenic progression or colonization upon transplantation, a key feature of bona fide SSCs (45, 46). Therefore, while our results demonstrate the feasibility of CRISPR/Cas9-mediated editing in a germline-relevant context, future studies incorporating primary SSCs or transplantation assays will be essential to validate germline transmission potential and physiological relevance.

SSCs exhibit a relatively slow *in vitro* doubling time. For mouse SSCs, it ranges between 3 to 4 days (46, 47). In various culture conditions, such as on laminin or feeder layers, reported doubling times range from 2.7 to 5.6 days. In our study, the doubling time was found to be 3.5 days, consistent with reports for SV40 large T-antigen-transformed lines, which prolong proliferation and sustain germline potential (44).

In transfection experiments, selectable markers such as antibiotic resistance or fluorescence facilitate tracking transfected cells. In this study, the Pac gene provided puromycin resistance, and an MTT assay determined 2 μg/mL for 48 h as optimal for selection. This is consistent with CRISPR studies in ESCs (48).

In the bacterial transformation stage, we used CaCl_2_-treated DH5α competent cells. Contrary to Froger and Hall (49), who reported success with 45 s heat shock, a 2-min heat shock yielded better transformation in our hands—likely due to strain-specific membrane composition (50, 51).

For SSC transfection, both viral and non-viral methods have been explored. Due to the immune risks of viral vectors, lipid-based chemical delivery methods are increasingly preferred (28, 52). While SSC genome editing via non-viral vectors is rare, lipofection-based approaches have shown high efficiency in ESCs (53). Our study is the first to use DNAfectin™ in SSCs, demonstrating effective CRISPR-Cas9-mediated editing.

We targeted exon 1 of the Tex15 gene with three sgRNAs. Although three different sgRNAs were designed to target exon 1 of the Tex15 gene, only Target 1 successfully induced detectable indel mutations confirmed by ICE analysis and yielding a 99% predicted KO score. The inability of Target 2 and Target 3 to generate edits may be attributed to several factors including low sgRNA efficiency, the presence of genomic polymorphisms or mismatches at the target loci, suboptimal PAM sequences, or steric hindrance due to local chromatin architecture. It has been previously shown that even single nucleotide mismatches within the seed region or near the PAM site can dramatically reduce CRISPR/Cas9 activity (54, 55). Moreover, chromatin accessibility plays a critical role in editing efficiency, as inaccessible loci can prevent Cas9 from binding and cleaving the target DNA (56–58). Thus, our findings highlight the importance of empirically testing multiple sgRNAs when targeting new genomic loci and suggest that pre-screening for chromatin openness and potential polymorphisms may enhance success rates.

Transfection efficiency is influenced by DNA: lipid ratio. Our results showed optimal editing at 1:3.5 and 1:5 DNA: DNAfectin ratios, with higher lipid ratios (1:7.5 and 1:10) reducing efficiency due to endosomal retention (59). At 1:3.5, indels were detected in all cells. Variation in editing rates was observed between subgroups due to differences in cell density, suggesting the need for further research.

Post-transfection, CRISPR editing may result in wild-type, heterozygous, biallelic mosaic, or homozygous mutations. For complete gene disruption, biallelic edits are required. We enriched for monoclonal SSCs to eliminate allele variability. From 20 single cells, one monoclonal line was established. ICE analysis confirmed the absence of wild-type alleles in the monoclonal line derived from a single cell, providing direct evidence of biallelic CRISPR-Cas9-mediated editing. The identification of two major indel variants—one at 50% and the other at 20% frequency—strongly suggests that both alleles of the target locus were independently edited, resulting in a heteroallelic genotype. This scenario is supported by the clonal nature of the cell population, which rules out intercellular variability and highlights intra-genomic diversity arising from double-strand break repair. The relatively high contribution of a single indel (50%) could indicate that this variant was more efficiently produced during non-homologous end joining (NHEJ), while the presence of a second variant at a lower frequency (20%) may reflect allelic bias in repair pathway engagement or repair fidelity. These data align with prior observations that even in single cells, the Cas9 complex can induce distinct mutations on each allele, depending on microenvironmental factors and chromatin accessibility. Given the single-cell clonal origin, this experiment emphasizes the necessity of allelic-level resolution in the interpretation of CRISPR outcomes, particularly in therapeutic settings where monoallelic vs. biallelic modifications can have significantly different phenotypic consequences. However, more clones would be necessary to confirm homozygosity.

Tex15 mutations are known to impair double-strand DNA repair and cause infertility (15). However, no previous CRISPR-based studies had targeted Tex15 directly. This study is the first to generate a Tex15 knockout monoclonal SSC line using DNAfectin-mediated CRISPR-Cas9 genome editing.

This study demonstrates that lipid-based, non-viral transfection using DNAfectin™ is a viable and effective method for CRISPR-Cas9-mediated genome editing in mouse spermatogonial stem cell lines. Optimization of transfection conditions, including DNA:lipid ratios and antibiotic selection, enabled efficient gene disruption of the Tex15 gene. The successful establishment of monoclonal edited cell lines further confirms the method's reliability. These findings pave the way for SSC-based transgenic research and therapeutic applications targeting male infertility and genetic diseases.

## Author's note

This article presents the results of the doctoral dissertation of Süheyla Yeşilbostan, conducted at the Department of Veterinary Genetics, Graduate School of Health Sciences, Ankara University, under the supervision of Bengi Çınar.

## Data Availability

The datasets generated and/or analyzed during the current study are not publicly available due to their ongoing use in a related research project, but they are available from the corresponding author upon reasonable request.
